# Individual differences in vagal regulation are related to testosterone responses to observed violence

**DOI:** 10.3389/fpsyg.2015.00019

**Published:** 2015-02-24

**Authors:** Eric C. Porges, Karen E. Smith, Jean Decety

**Affiliations:** ^1^Institute of Aging, Cognitive Aging and Memory Clinical Translational Research Program, University of Florida, Gainesville, FL, USA; ^2^Department of Psychology, University of Chicago, Chicago, IL, USA; ^3^Department of Psychiatry and Behavioral Neuroscience, University of Chicago, Chicago, IL, USA

**Keywords:** pain, respiratory sinus arrhythmia, testosterone, violence, challenge hypothesis, parasympathetic, autonomic nervous system

## Abstract

Observing violent content has been hypothesized to facilitate antisocial behaviors including interpersonal violence. Testosterone is released in response to perceived challenges of social status, often followed by an increase in aggressive behaviors and physiological activation. Prior investigations evaluating the impact of observing violence on autonomic function have focused on sympathetic measures of arousal. Measurement of parasympathetic nervous system (PNS) activity has been neglected, although reduced PNS activity has been associated with antisocial behavior. Consistent with a hierarchical model of the autonomic nervous system (i.e., polyvagal theory), individual differences in PNS activity reflected in respiratory sinus arrhythmia (RSA) were hypothesized to have an inhibitory impact on sympathetic and hormonal reactivity in subjects who were observing a violent video. Autonomic data (i.e., electrodermal activity (EDA), heart rate, and RSA) were collected from forty adult males prior to and while viewing violent sports or a control video. Pre- and post-video saliva samples were assayed for cortisol and testosterone. Participants who viewed the violent video showed increased sympathetic activity compared to controls. In contrast to the sympathetic reactivity to the violent video, there were no significant RSA changes in response to the stimuli, suggesting that viewing violent sports selectively increases sympathetic activity without eliciting PNS withdrawal. However, within the group viewing the violent video, participants with lower RSA during baseline and the observation of violent videos, responded with greater increases in salivary testosterone, suggesting that high parasympathetic tone dampens testosterone reactivity. These individual differences in response to observed violence, associated with higher RSA, may account for some of the improved health, growth, and restoration outcomes across the lifespan, that this segment of the population benefits from.

## INTRODUCTION

The link between observing violence and the expression of violent behavior has a long history dating to the Victorian era (Paik and Comstock, 1994). The prevalence and ubiquity of violence in the modern media has spurred considerable interest, debate and public concern about its potential harm in forums ranging from scientific literature to Supreme Court and congressional hearings (Anderson and Bushman, 2001; Hall et al., 2011). Despite these concerns and mixed results from empirical research, it seems clear that there is a large range of individual differences in responses to observed violence, with the great majority of individuals not committing violent acts after observing violence. Given such individual variation, investigating individual differences in physiological responses to violent media may help characterize vulnerabilities that could manifest as antisocial behavior.

Testosterone has been the focus of research because of its putative role in male aggression. However, the exact role testosterone plays in facilitating aggressive behavior has been widely debated (Eisenegger et al., 2011; Josephs et al., 2011; van Honk et al., 2011b). Prior work has linked testosterone associated aggressive behaviors with functions that support reproductive/mating opportunities, such as gaining territory or improving social status. However, testosterone release is not without cost in the form of increased metabolic demands and immune system suppression (Boonekamp et al., 2008). The challenge hypothesis proposes that, because testosterone release can be so metabolically costly, an organism must balance the physiological and behavioral consequences of high testosterone levels to meet their environmental demands (Archer, 2006). As a result of this cost, when a direct, proxied, or symbolic opportunity to improve reproductive standing is not at stake, testosterone levels should not be responsive to a given situation. This hypothesis was originally used to describe the behavior of avian species, which exhibit slow seasonal changes in testosterone levels. Spikes in testosterone were detected when direct breeding opportunities or social instability occurred (Wingfield et al., 1990). The theory has since been tested in primates and used to predict behavior in humans (Archer, 2006), with research suggesting that aggression related to territoriality and dominance releases testosterone, while defensive aggression does not result in a similar release of testosterone. This led to the hypothesis that social elicitors of testosterone release in human males are either threats to social status (Josephs et al., 2011) or anticipation of reproductive opportunities (Wingfield et al., 1990). Behaviors that influence human social status or present a challenge to social status may take many direct and indirect forms. For example, a social interaction in which status is challenged may range from active participation in a physical sport, to competing for desirable jobs, to a game of chess (Mazur et al., 1992). The consequences of these challenges (i.e., winning or losing) on testosterone levels are less clear, with some studies reporting a positive relationship while others reporting no relationship at all, and the consequences often being depending on individuals’ own subjective perceptions and evaluations of what constitutes a win. For example, a technical loss may be considered a win if the individual expected a worse outcome (Costa and Salvador, 2012; Jiménez et al., 2012; van der Meij et al., 2012).

Testosterone influences individual targets and networks in the central nervous system that are known to be involved in the perception of threat related social stimuli. Both endogenous and exogenous testosterone appear to modulate amygdala response to fear and anger expression (Derntl et al., 2009), a neural structure involved in emotion perception and processing with a particularly sensitivity to threat related stimuli (Ohman, 2005; LeDoux, 2007). Additionally, endogenous testosterone levels are associated with prefrontal cortex and amygdala functional connectivity in response to affective social stimuli (Volman et al., 2011). Alterations in medial prefrontal cortex (mPFC) activity have been proposed to be one of the primary neurobiological mechanisms relating endogenous testosterone levels to aggressive behaviors (Mehta and Beer, 2010). For example, previous studies have found that circulating testosterone levels are related to ventromedial prefrontal cortex responses to anger in males (e.g., Stanton et al., 2009).

Recent work has demonstrated that the dorsal aspect of the mPFC (Napadow et al., 2008) is correlated with parasympathetic activity, and the mPFC generally may serve as a final common pathway linking affect with autonomic system response (Lane et al., 2009). The parasympathetic branch of the autonomic nervous system, via the myelinated vagus nerve, exerts inhibitory control of both behavior and cardiac activity (Porges, 2007) and has been shown to interact with the HPA axis (Ulrich-Lai and Herman, 2009), with stimulation of the vagus reducing HPA axis reactivity to corticotrophin releasing hormone (O’Keane et al., 2005). Throughout the present experiment, we measured heart rate and respiratory sinus arrhythmia (RSA), an index of vagal modulation of the parasympathetic system (Porges, 2007). Research suggests that reduced resting RSA may result in an inability to down regulate threat detection, potentially leading to inappropriate interpretations of social cues (Thayer and Lane, 2000). Atypical regulation of RSA is associated with many psychiatric disorders including major depressive disorder and generalized anxiety disorder (Kemp et al., 2012), populations with high levels of aggression, such as perpetrators of violent domestic abuse (Umhau et al., 2002) and delayed recovery of RSA after a moderate exercise has been reported in women with a history of trauma (Dale et al., 2009).

In the present study, we collected measures of heart rate, RSA, electrodermal activity (EDA; a measure of sympathetic reactivity), salivary testosterone, and salivary cortisol while participants viewed a violent sport competition to examine individual differences in patterns of physiological responding to observed violence. We restricted our study sample to males because the challenge hypothesis, which shaped our research question, is based on male testosterone responses and because the past literature on testosterone responses are more consistent in males than in females (Josephs et al., 2011). In keeping with previous work on viewing acts of violence (Palomba et al., 2000), we predicted that participants would exhibit increased heart rate and EDA, and, possibly, increased cortisol. Bridging research on vagal regulation and challenge hypothesis (Wingfield et al., 1990), we predicted that testosterone release should not occur in those participants with relatively higher parasympathetic tone (as indexed by RSA) as they should not perceive a violent video as posing a threat to their social status. However, given the more reactive and defensive state associated with reduced parasympathetic tone (Porges, 2007), relatively lower RSA should increase the tendency to perceive social status threat and this should in turn should result in increased testosterone release. Finally, we examined whether individual differences in RSA reactivity to observing a violent competition were associated with differential patterns of neuroendocrine (cortisol, testosterone) activation.

## MATERIALS AND METHODS

### PARTICIPANTS

Males aged 18–35 (*n* = 43) were recruited from the University of Chicago campus and compensated with course credit or payment for their participation in the study. Participants were not screened for current health status, but were instructed to avoid alcohol for 12 h prior and nicotine for 1 h prior to participation. Heart rate and RSA were within normative range for this population (Byrne et al., 1996). Participants’ written informed consent was obtained, and this study was approved by the Institutional Review Board at the University of Chicago. Three subjects were dropped from analysis due to equipment problems, leaving 40 participants, 20 each randomly assigned to control or experimental conditions.

### STIMULI

Participants were randomly assigned to one of two groups. The experimental group was shown a 28-min video of a complete Mixed Martial Arts (MMA) fight (UFC 86 Forrest Griffin vs. Quinton “Rampage” Jackson, 2008). MMA is a full contact combat sport similar to boxing that involves the use of martial arts techniques. The selected fight had closely matched contestants and was judged as being extremely close, continuing for a full five rounds with no clear winner in contemporary press reports (Brookhouse, 2008) and the outcome being decided by a judge’s decision that was not shown to subjects. The selection of a fight without an obvious winner and with contestants that unfamiliar to the subjects was done to eliminate any hormonal changes induced by rooting for the winner or loser in a sporting event (Bernhardt et al., 1998). The control group viewed a 28-min non-arousing video on environmental building methods selected to mimic baseline controls in similar studies (Wirth and Schultheiss, 2006).

### PROCEDURE

Participants abstained from alcohol for 12 h and food, caffeine, and nicotine for 1 h prior to the experiment to prevent contamination of the saliva samples (Salimetrics^©^, 2009; Poll et al., 2007). Upon arrival, participants were brought to a private study room, briefed on the protocol, and informed consent was acquired. Electrodes were attached for EDA and electrocardiography (ECG), and participants were asked to give a practice saliva sample to expose them to the procedure prior to experimental sample collection. The experimenter then left the participants in the room to complete a series of questionnaires administered on a computer. Completion of these questionnaires was used as a non-arousing task for participants during 30 min of sitting in a still and quiet environment, necessary to bring physiological measures to baseline (Lam et al., 2009). Baseline measures were taken while completing non-arousing questionnaires, since it has been argued that having participants perform a low level cognitive task may provide a better baseline than sitting quietly as it leaves less room for variance in the activity they are engaged in (Jennings et al., 2007).

After the 30-min period, a baseline salivary hormone sample was collected and placed immediately into a -20°C freezer. Autonomic measures, which act on a more rapid time course of seconds and typically are left for ∼5 min to stabilize, had also stabilized at this point. Participants were then left alone to watch either the fight or documentary. After the video, a second saliva sample was collected, electrodes were removed, and participants in the experimental condition were asked to answer two questions regarding their rooting behavior (“which fighter were you rooting for?” and “which fighter did you think won the fight?”). These measures were used to confirm that participants’ perceptions of who won and who they wanted to win did not influence their physiological responses to the fight by running correlations between participants’ ratings and the physiological measures. These correlations were not significant (see **Table 1**).

**Table 1:** Non-significant correlations between physiological measures and winning and rooting ratings for participants who watched the fight.

Physiological measure		Rooting	Won
RSA baseline	Pearson correlation Sig (2-tailed) *N*	–0.350 0.130 20	-0.167 0.482 20
RSA Epoch 1	Pearson correlation Sig (2-tailed) *N*	-0.252 0.283 20	–0.173 0.466 20
RSA Epoch 2	Pearson correlation Sig (2-tailed) *N*	–0.152 0.522 20	–0.312 0.181 20
RSA Epoch 3	Pearson correlation Sig (2-tailed) *N*	-0.226 0.338 20	-0.218 0.357 20
RSA Epoch 4	Pearson correlation Sig (2-tailed) *N*	-0.260 0.268 20	-0.124 0.573 20
RSA Epoch 5	Pearson correlation Sig (2-tailed) *N*	-0.276 0.240 20	-0.068 0.776 20
Heart rate baseline	Pearson correlation Sig (2-tailed) *N*	-0.446 0.049 20	0.057 0.811 20
Heart rate Epoch 1	Pearson correlation Sig (2-tailed) *N*	0.300 0.198 20	0.077 0.746 20
Heart rate Epoch 2	Pearson correlation Sig (2-tailed) *N*	0.295 0.207 20	0.158 0.506 20
Heart rate Epoch 3	Pearson correlation Sig (2-tailed) *N*	0.357 0.122 20	0.051 0.831 20
Heart rate Epoch 4	Pearson correlation Sig (2-tailed) *N*	0.311 0.181 20	0.051 0.831 20
Heart rate Epoch 5	Pearson correlation Sig (2-tailed) *N*	0.258 0.272 20	0.066 0.876 20
EDA baseline	Pearson correlation Sig (2-tailed) *N*	0.067 0.779 20	-0.102 0.668 20
EDA Epoch 1	Pearson correlation Sig (2-tailed) *N*	0.029 0.905 20	0.282 0.229 20
EDA Epoch 2	Pearson correlation Sig (2-tailed) *N*	-0.057 0.810 20	0.071 0.765 20
EDA Epoch 3	Pearson correlation Sig (2-tailed) *N*	-0.144 0.544 20	0.157 0.508 20
EDA Epoch 4	Pearson correlation Sig (2-tailed) *N*	-0.012 0.961 20	0.170 0.766 20
EDA Epoch 5	Pearson correlation Sig (2-tailed) *N*	0.021 0.929 20	0.309 0.110 20
Pre-video cortisol	Pearson correlation Sig (2-tailed) *N*	0.181 0.472 18	-0.098 0.698 18
Post-video cortisol	Pearson correlation Sig (2-tailed) *N*	0.204 0.417 18	-0.159 0.530 18
Residual change cortisol	Pearson correlation Sig (2-tailed) *N*	0.093 0.714 18	-0.164 0.514 18
Pre-video testosterone	Pearson correlation Sig (2-tailed) *N*	0.108 0.668 18	-0.138 0.585 18
Post-video testosterone	Pearson correlation Sig (2-tailed) *N*	0.214 0.394 18	-0.159 0.528 18
Residual change testosterone	Pearson correlation Sig (2-tailed) *N*	0.239 0.314 18	-0.087 0.730 18

### AUTONOMIC NERVOUS SYSTEM MEASUREMENTS AND QUANTIFICATION PROCEDURES

All physiological measurements were recorded using AcqKnowledge data recording software (version 3.8.1), running on a Windows XP computer, connected to a set of Biopac © amplifiers connected to a Biopac © MP 100 A A/D digitization system (digitizing signals at 1 kHz; Biopac © Systems, Inc., Santa Barbara, CA, USA). Electrodes were left undisturbed for 5 min before data was collected to ensure they reached stable impedance. EDA, heart rate, and RSA were collected throughout the entire experiment. Data from the videos were split into 5.6 min segments to correspond with the five rounds of the fight and 5.6 min of data were extracted from the questionnaire period as baseline.

### ELECTRODERMAL ACTIVITY

Electrodermal activity was collected using a pair of Ag/AgCl electrodes filled with Biopac © isotonic electrode gel from the distal phalanges of digits II and III of the left hand (Stern et al., 2001). AcqKnowledge was used to extract and count the skin conductance responses (SCRs), which reflect sympathetic nervous system arousal with a time course of 3–5 s, from the EDA recording using its automated SCR detector with an SCR threshold of 0.02 μmho (Dawson et al., 2000). This analysis was manually supervised to ensure proper SCR identification and the exclusion of artifacts.

### HEART RATE AND RSA

A Biopac mp100 was used to collect the ECG signal. The ECG was sampled at 1 kHz and a 60 Hz notch filter and a 0.5–35 Hz finite impulse response (FIR) filter (Ruha and Nissila, 1997) were applied. Inter-beat intervals (IBIs) series, as defined by R–R intervals in milliseconds, were extracted using supervised R-wave peak detection in AcqKnowledge 4.1 (Biopac^©^, 2000). IBI data were edited as previously described (Lewis et al., 2012) in order to remove artifacts that might confound the quantification of periodic components. Editing and heart rate variability quantification were performed with CardioEdit and CardioBatch programs (Brain-Body Center, University of Illinois at Chicago, IL, USA). The IBI series were linearly interpolated and filtered to remove spontaneous (0.12–0.40 Hz) breathing for RSA. A lower threshold of 0.12 Hz was utilized, as reports of healthy participants exhibiting respiration rates slower than 0.15 Hz are not uncommon (Denver et al., 2007; Saboul et al., 2014). The result is mathematically equivalent during steady state conditions to spectral analysis. This method is not moderated by respiration and conforms to the assumptions necessary for parametric statistics (Lewis et al., 2012). Data for RSA were quantified for sequential 30 s epochs, and data for heart rate were quantified in IBIs within each 5.6 min condition (i.e., baseline or round). Measures of average heart rate and average RSA were quantified for each condition.

While there is debate over whether respiration rate should be controlled for accurate measurement of RSA (Quintana and Heathers, 2014), the relationship between heart rate and RSA implemented here, is not moderated by respiration (Lewis et al., 2012). Furthermore, subjects were given no instruction with regard to their respiration rate as there is evidence that paced breathing instruction could add an additional cognitive demand by inducing dual attentional demands (Quintana and Heathers, 2014), and as result influencing the processing of the stimuli.

### HORMONAL SAMPLE COLLECTION

Salivary testosterone and cortisol were obtained through saliva samples collected using un-stimulated passive drool techniques (Roney et al., 2007; Fairchild et al., 2008). This involved participants pooling saliva in their mouth for a minute and then drooling passively through a straw into a 2 mL Cryovial (Salimetrics^©^, 2009) until the vial was full. Samples were frozen immediately in a -20°C freezer kept on site and then moved to a -80°C freezer until analysis. After completion of data collection, saliva samples were thawed and hormonal assays were performed on the saliva for both testosterone and cortisol using Salimetrics © enzyme immunoassay kits for testosterone and cortisol as instructed (Salimetrics^©^ 2011). Inter-assay CV value for cortisol was 3.56% and for testosterone was 2.63%. Intra-assay CVs for cortisol were 3.92 and 5.84% for each plate and for testosterone were 6.10 and 6.04% for each plate. Both testosterone and cortisol levels were within already published ranges for similar populations (Burnham et al., 2003; Wirth and Schultheiss, 2006; Murphy et al., 2010; Pilgrim et al., 2010; Randler et al., 2012). Due to insufficient amounts of saliva, four participants’ saliva samples were excluded. This left a total of 36 measurements each for testosterone level, measured in pg/ml, and cortisol level, measured in μg/dl, with 18 measurements in the control group and 18 in the fight group.

### STATISTICAL ANALYSES

In order to examine the autonomic and hormonal effects of viewing violence, data were screened for outliers using the interquartile range multiplier approach (Tukey, 1977; Hoaglin, 1986), with a multiplier if 2.2 (Hoaglin and Iglewicz, 1987), using this approach, no outliers were identified. We first ran repeated measures ANOVAs for the autonomic measures (EDA, heart rate, RSA) and hormonal measures (cortisol and testosterone) to examine differences in autonomic and hormonal responses between the fight and control group over time. In addition, correlations were performed with pre and post-stimuli and change (using unstandardized residuals where appropriate) in hormonal measures and RSA in the fight group to assess any potential relationships between parasympathetic reactivity and hormonal responsivity to viewing violence. Effect sizes were also calculated for all the analyses using Cohen’s d. SPSS (v. 18.0, SPSS Inc., Chicago, IL, USA) was used for statistical analyses.

## RESULTS

### ELECTRODERMAL ACTIVITY

**FIGURE 1:**
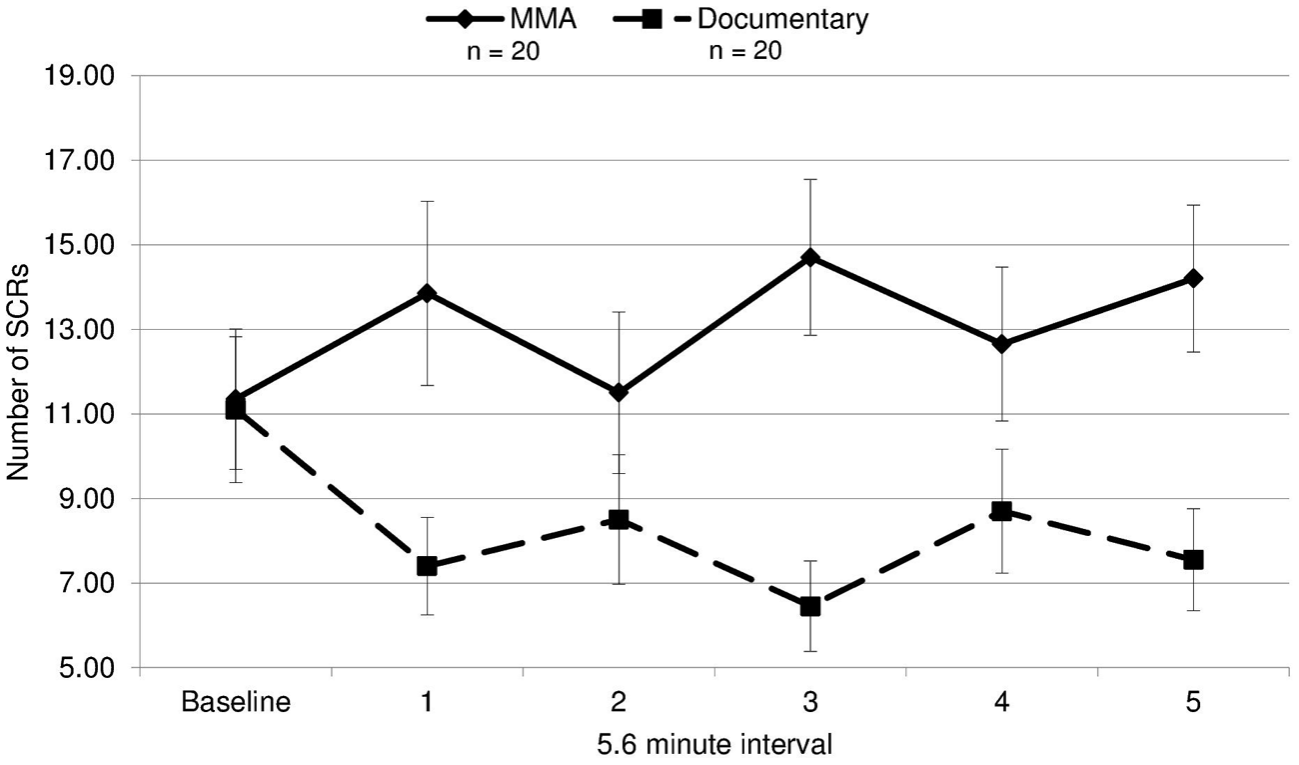
**Electrodermal activity.** The mean number of skin conductance responses (SCRs) during each 5.6 min interval for the fight and the control documentary (error bars represent SD).

To analyze group differences in EDA, a repeated measures ANOVA was performed: 2 (group: experimental/control) × 6 (interval: 5.6 min from baseline and five 5.6 min segments from the fight corresponding with each round of the fight). There was a main effect for EDA [*F*(1,19) = 5.91, *p* = 0.020, *d* = 0.44], indicating that the group observing the violent sporting event had significantly more SCRs. Moreover, there was a significant group by interval effect [*F*(3.53,19) = 4.59, *p* = 0.003, ε = 0.71^1^; **Figure 1**]. This interaction was characterized not only by the group observing violence having increased SCRs overall but also exhibiting round-specific differences compared to the group viewing the documentary. Uncorrected contrasts identified rounds 1 [*F*(1,19) = 5.91, *p* = 0.020], 3 [*F*(1,19) = 12.40, *p* = 0.001], and 5 [*F*(1,19) = 8.98, *p* = 0.005] as significantly different from baseline by group. This was not the case for rounds 2 [*F*(1,19) = 1.13, *p* = 0.295] and 4 [*F*(1,19) = 2.71, *p* = 0.108].

**FIGURE 2:**
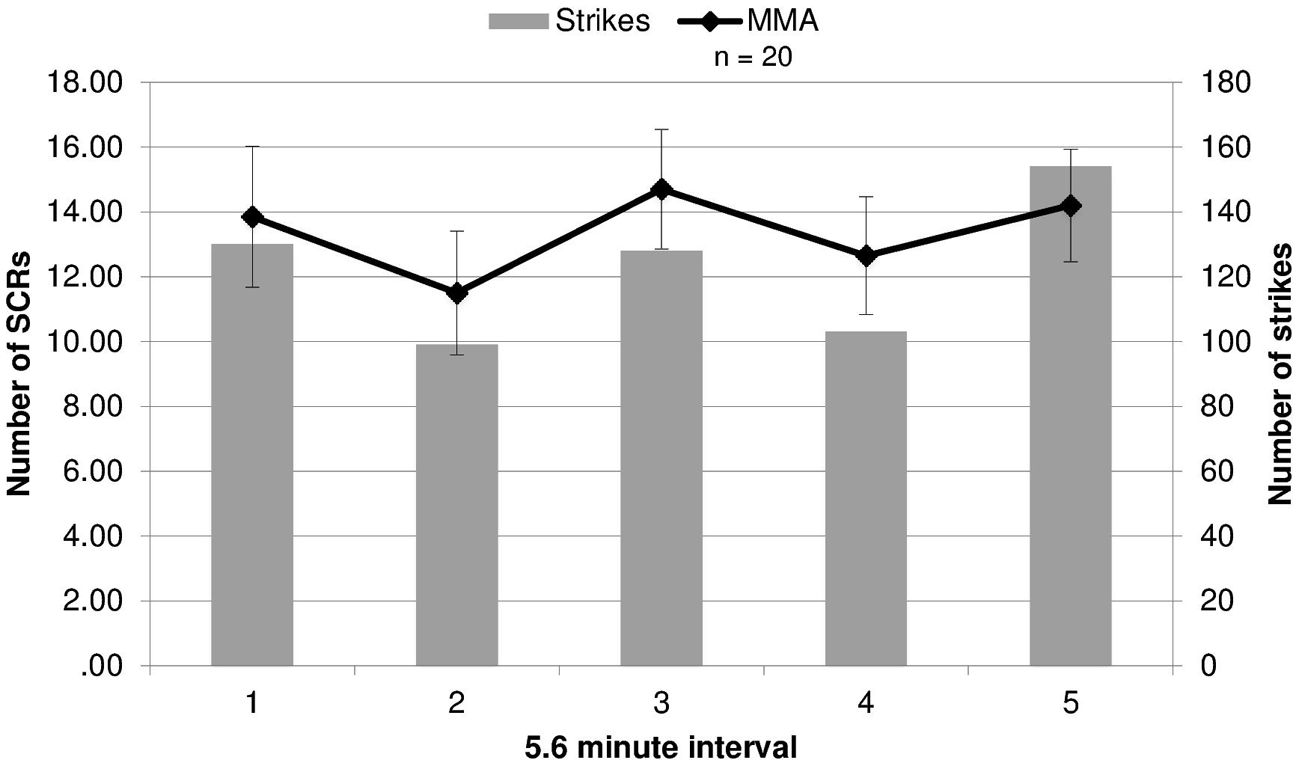
**Electrodermal activity responses compared to strikes during fights.** The number of strikes and number of skin conductance responses (SCRs) during each round of the fight (error bars represent SD).

Because SCR response differed by round, we evaluated whether the round by round differences in SCRs were related to round specific quantitative differences in physical violence. Different amounts of violence between rounds might drive the differing EDA responses in viewers. This was tested by contrasting the number of strikes and maneuvers during each round of the MMA fight, quantified using an independent professional service which provides statistics for MMA and boxing events (, UFC 86: Jackson vs. Griffin), with the number of SCRs for each round. The rounds in which SCRs did not differ significantly from the baseline by group (rounds 2 and 4) contained the fewest strikes (**Figure 2**). This quantitative index of violence during each round mapped onto participants’ EDA response to viewing the fight. Additionally, a correlation analysis was run between average SCRs for each round and total number of strikes, bordered on significance (*r* = 0.82, *p* = 0.090).

### HEART RATE AND RSA

**FIGURE 3:**
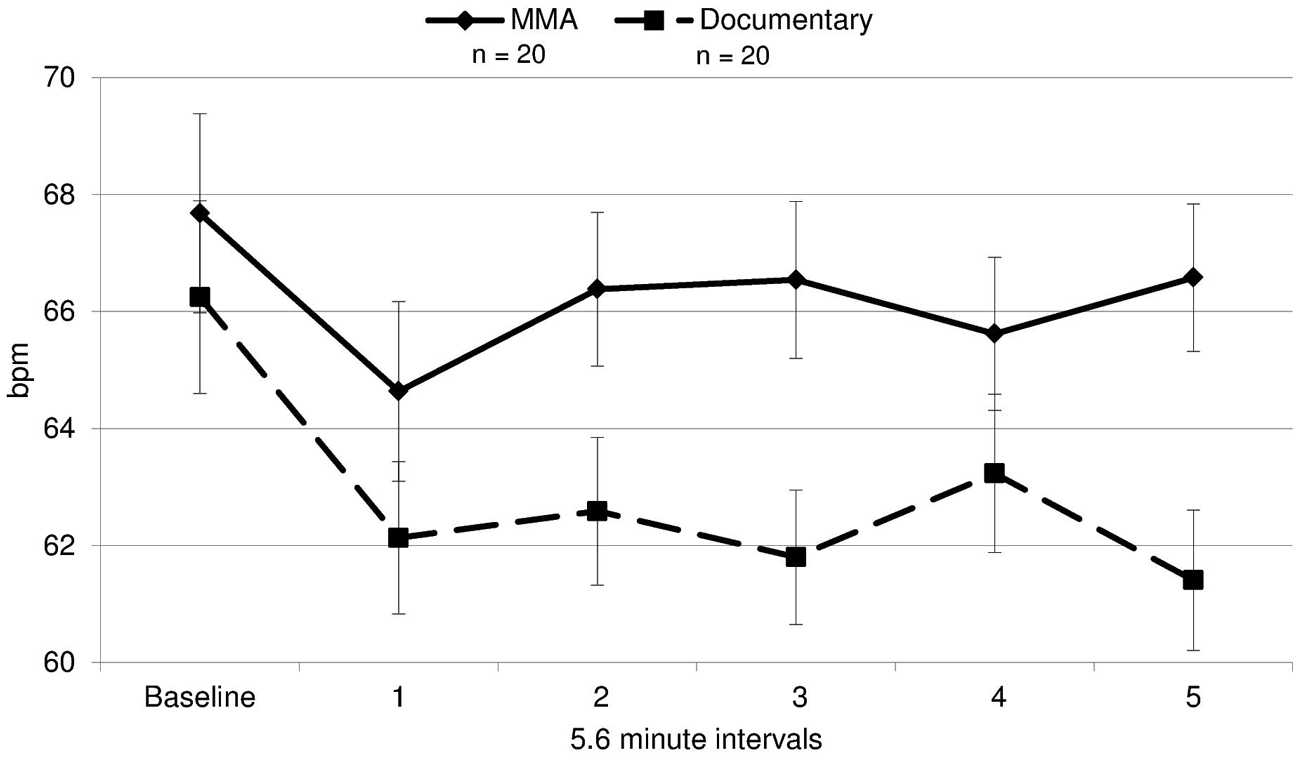
**Heart rate.** Heart rate in beats per minute (bpm) during each time interval for each group for the fight and control group (error bars represent SD).

**FIGURE 4:**
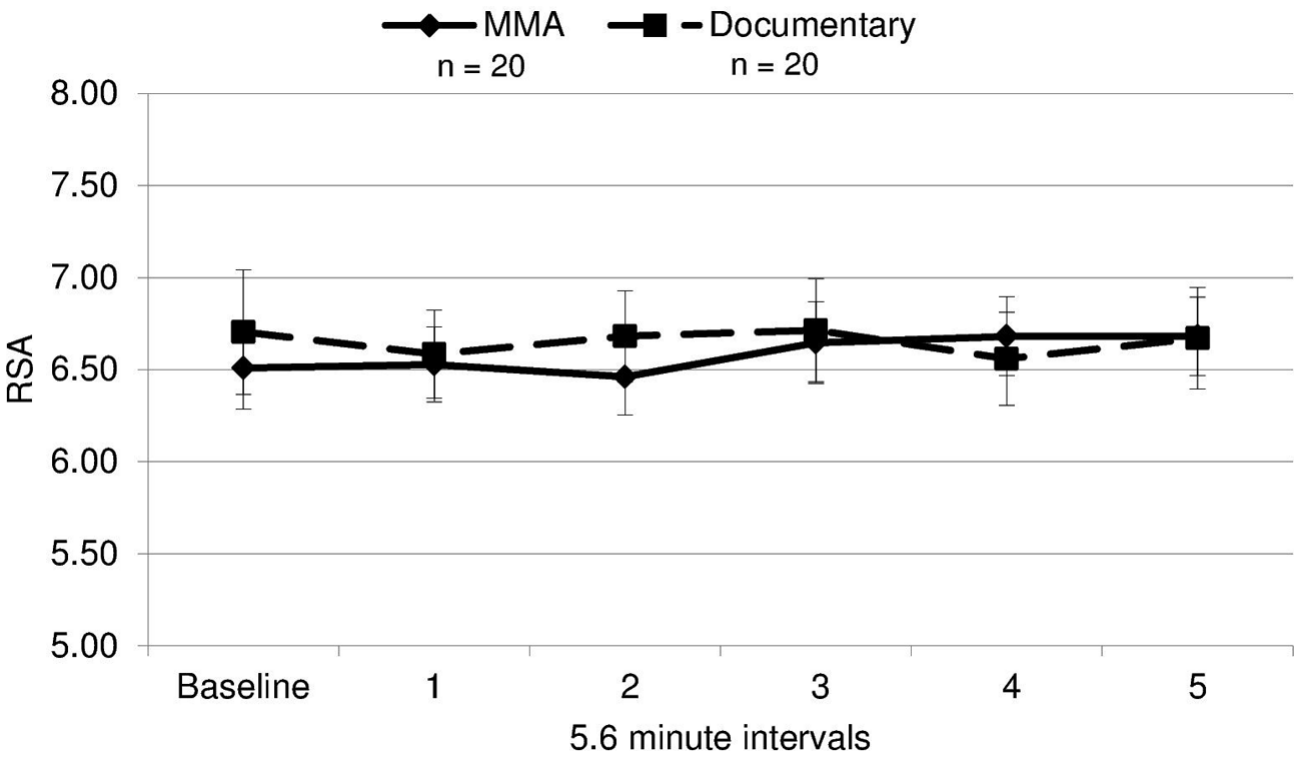
**RSA means.** Respiratory sinus arrhythmia (RSA, i.e., a measure of parasympathetic nervous system activity) during each interval for each group (error bars represent SD).

A 2 (condition: fight/control) × 6 (six 5.6 min intervals) repeated measures ANOVA was performed on both RSA and heart rate data to determine group differences and within-subject differences across time. For heart rate, a main effect of time [*F*(3.30,19) = 14.321, *p* = 0.000] and a near significant effect of group [*F*(3.30,19) = 3.26, *p* = 0.079] were identified. There was a significant group by condition interaction for heart rate [*F*(3.30,19) = 5.04, *p* = 0.002, ε = 0.66]^1^. The differences between groups were amplified as the study progressed (**Figure 3**), with heart rate increasing in the fight group and decreasing in the control group. Uncorrected contrasts identified significant differences between groups during rounds one and 3 [*F*(1,19) = 8.59, *p* = 0.006], three and 4 [*F*(1, 19) = 7.54, *p* = 0.009], and 4 and 5 [*F*(1,19) = 14.13, *p* = 0.001]. In addition, uncorrected contrasts identified significant differences between baseline and rounds three by group [*F*(1,19) = 6.07, *p* = 0.018] and 5 [*F*(1,19) = 11.43, *p* = 0.002]. In contrast, as illustrated in **Figure 4**, RSA remained relatively stable through the experiment and did not exhibit group differences across baseline or the five rounds [*F*(1,19) = 0.05, *p* = 0.834, *d* = 0.004].

### HORMONAL MEASURES

In order to discern if there were any significant group differences in changes in testosterone and cortisol, a 2 (group: MMA/control) × 2 (pre and post hormone levels) repeated measures ANOVA was performed for both testosterone and cortisol. There was a main effect of time for cortisol [*F*(1,34) = 13.097, *p* = 0.001] but not for testosterone [*F*(1,34) = 1.192, *p* = 0.283]. Neither testosterone [*F*(1,17) = 0.45, *p* = 0.509, *d* = 0.17] nor cortisol [*F*(1,17) = 2.81, *p* = 0.103, *d* = 0.40] exhibited any significant differences between groups, suggesting that there were no significant increases in either measure in response to viewing the fight compared to the documentary. The medium effect size for cortisol (*d* = 0.40) suggests that the non-significant finding for cortisol may be a result of the low power (*n* = 18 per group) and should be investigated in future research.

**FIGURE 5:**
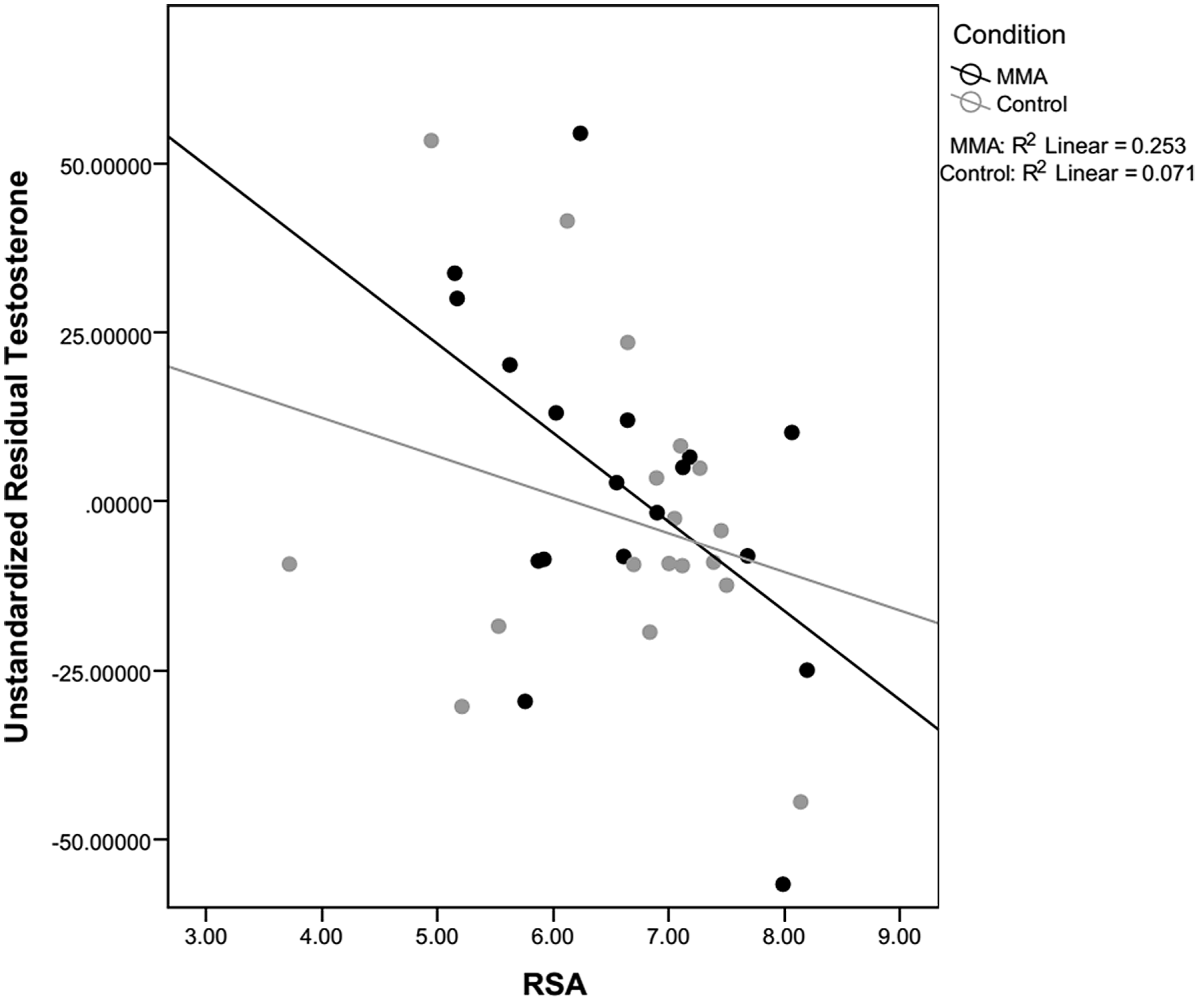
**Correlation between RSA and testosterone.** Correlations between residual testosterone and respiratory sinus arrhythmia (RSA) level for the fight and control conditions. Only correlations for the fight were significant.

For both testosterone and cortisol, change scores were calculated by taking post video level minus pre video level. Unstandardized residuals were then calculated by running a regression analysis using post-fight levels as the dependent variable and raw change score as the independent variable and saving the unstandardized residuals. While residual change is often calculated by regressing the post measure on pre measure, we used the more conservative method, regressing change from pre measures to post measures on pre measures. Using unstandardized residuals in place of raw change scores provides a more accurate depiction of change by accounting for variance in initial baseline levels between subjects, see **Table 2** (Mehta and Josephs, 2006). To examine whether there was a relationship between hormone responsivity to the fight and RSA, exploratory correlations between testosterone and cortisol unstandardized residuals and RSA for the fight and control group separately. Because RSA did not change in response to the fight, RSA was averaged over the six epochs (baseline and the five rounds) and this value was used in the correlations. A statistically significant correlation for the fight group was observed between the unstandardized residual for testosterone and RSA [*r*(16) = -0.503, *p* = 0.033; see **Figure 5**]. The correlation between the unstandardized residual for cortisol and RSA for the fight group was not statistically significant [*r*(16) = -0.361, *p* = 0.142], and there were no significant correlations between the unstandardized residuals for cortisol and testosterone and RSA in the control group [Testosterone: *r*(16) = -0.266, *p* = 0.286; Cortisol: *r* (16) = 0.242, *p* = 0.333]. The significant negative correlation between RSA and testosterone responses suggests that viewers with lower levels of RSA throughout the baseline condition and the fight exhibited greater increases in testosterone while viewing the fight.

**Table 2:** Summary of descriptive statistics for hormonal measures.

	Fight		Documentary	
	*M*	SD	*M*	SD
Pre-video cortisol	0.24 μg/dl	0.17 μg/dl	0.18 μg/dl	0.07 μg/dl
Post-video cortisol	0.20 μg/dl	0.12 μg/dl	0.13 μg/dl	0.05 μg/dl
Pre-video testosterone	150.8 pg/ml	54.1 pg/ml	141.3 pg/ml	52.6 pg/ml
Post-video testosterone	146.7 pg/ml	48.1 pg/ml	135.0 pg/ml	43.2 pg/ml
Residual cortisol	0.01 μg/dl	0.05 μg/dl	–0.01 μg/dl	0.04 μg/dl
Residual testosterone	2.4 pg/ml	25.2 pg/ml	–2.4 pg/ml	23.4 pg/ml

## DISCUSSION

This study investigated individual differences in autonomic and hormonal responses to viewing violence. Specifically, whether viewing violence results in a release of testosterone, and whether individuals’ testosterone response to observed violence is modulated by individual differences in autonomic state. Research has previously demonstrated testosterone release in males in anticipation of conflicts that influence social status and reproductive opportunities (Archer, 1991). We predicted that violent stimuli without consequences for an individual’s social status and/or reproductive opportunities generally should not elicit a testosterone response in a healthy population. If it did, it would be most likely in participants with lower parasympathetic tone (as indexed by RSA). The polyvagal theory (Porges, 1995) predicts that, as a physiological state, relatively lower RSA is associated with a more reactive, defensive state. A testosterone response may provide evidence that a physiologically defensive individual may have experienced this violent video as a challenge to their status (Archer, 2006; Eisenegger et al., 2011; Josephs et al., 2011; van Honk et al., 2011a).

The findings of this study support our predictions. Participants in the experimental condition displayed no significant increases in testosterone as compared to the control group. While participants did exhibit increased EDA and heart rate in response to viewing violent stimuli, interestingly, they did not demonstrate any change in vagal tone measured by RSA. In addition, there was also no significant increase overall in cortisol in participants after viewing the fight, indicating that the viewing the fight was not perceived as a stressor, as cortisol is also associated with increased reactivity and stress mobilization (Foley and Kirschbaum, 2010).

While this study did not find an overall increase in testosterone in individuals viewing the fight, there was a negative correlation between RSA and testosterone change to viewing the fight, indicating that individuals with lower levels of RSA throughout the study had greater increases in testosterone in response to viewing the fight. This finding suggests that individuals with low baseline vagal tone may be more vulnerable to perceiving observed violence as a challenge or threat to their status than those outside of the low range of RSA. It has been reported that low RSA is a risk factor for social and emotional regulatory disorders. Low levels of baseline RSA are associated with varying forms of social and emotional disorders. For instance, women with a history of abuse, borderline personality disorder patients, and patients on the spectrum for autistic disorders (Porges, 2011) have been associated with decreased RSA and atypical regulation of RSA during task demands. In addition, children with a history of abuse and lower baseline levels of RSA are at a greater risk for developing conduct problems than children with a history of abuse but higher baseline levels of RSA (Katz, 2007). Low baseline RSA in infants has been associated with increased regulatory problems during infancy and behavioral difficulties later in childhood (Porges, 2011). These findings indicate that while lower levels of cardiac vagal tone, an index of central nervous system regulation, are linked with increases in testosterone to viewing violence suggesting a possible misperception of the observed violence as a direct threat to their status. These individuals may represent an already susceptible population, with associated difficulties in social interactions and emotion regulation. It is important to note, that as this study focused on individual differences in physiological responses to viewing violence and did not measure behavior after viewing the fight, statements about possible behaviors as a result of physiological state are purely speculative and should be further tested.

This study is one of the first to examine the complex interaction among components of the autonomic nervous system while viewing violence, incorporating both an index of parasympathetic activity (RSA), and measures of sympathetic responses (EDA). In the population of young men studied here, the sympathetic arousal exhibited while viewing violence would not be likely to produce increased reactivity and increased mobilization because the observed increase in sympathetic nervous system activity was not coupled with the withdrawal of the vagal brake, which serves to inhibit physiological arousal (Porges, 2007). The complementary withdrawal of parasympathetic tone and increase of sympathetic is characteristic of mobilization in response to a threat and there was no parasympathetic withdrawal observed. In addition, the lack of overall increases in testosterone levels suggests that observing violence alone is not sufficient to elicit testosterone release.

Due to the exploratory nature of this study, there are several limitations that need to be acknowledged. The first, and most importantly, is the relatively small sample size for the correlation analyses performed on the fight group (*n* = 18). Additionally, the correlation analyses in this study were exploratory in nature and need to be replicated in future studies. The generalizability of the population is also limited as participants were all college students, a population exposed to greater levels of chronic stress, which could affect physiological responses (Pruessner et al., 1999; Wüst et al., 2000; Dickerson and Kemeny, 2004). While this study was designed to investigate within-subject changes, the lack of normative responses to this novel paradigm limited the ability to interpret the findings. Future research should establish the reproducibility of these findings and account for attentional variance by utilizing a within-subjects rating of the stimuli. Additionally, the inclusion of “control” non-violent stimuli (e.g., tennis) that are comparably competitive and exciting would be informative.

Factors capable of influencing emotion regulation, including the role of viewing violence, and the physiological consequences of such experiences are only now becoming apparent. In the context of understanding the effects of observations of violence, future research should continue to investigate the possible role of changes in testosterone, as well as the potentially protective role of the parasympathetic nervous system (PNS). In addition, future studies should employ larger sample sizes and a broader range of individual differences and experiences, including gender differences in the effects of violent stimuli on endocrine and behavioral responses.

## CONCLUSION

This study examined physiological and hormonal responses to viewing violent stimuli, and found that in an undergraduate population, individuals only show increases in sympathetic nervous system activity, with no parasympathetic changes. However, and more importantly, in individuals with low baseline levels of RSA, a physiology already associated with vulnerability to psychopathology, observing violence elicited a greater increase in testosterone compared to those with higher levels of RSA. This may suggest that this already vulnerable population may be at risk for antisocial and aggressive behaviors after viewing violence. Furthermore, the testosterone release shown here may have additional consequences with regard to their perception and evaluation of social stimuli. The consequences of this across the lifespan, could may be an increased likelihood of conflict and the associated socioemotional consequences. Additional research utilizing wider age range, larger populations and explicit measures of post viewing behaviors is important to confirm and expand on this finding.

## Conflict of Interest Statement

The authors declare that the research was conducted in the absence of any commercial or financial relationships that could be construed as a potential conflict of interest.

## Footnotes

1 Degrees of freedom were adjusted using the Greenhouse-Geisser correction for violations of sphericity.
